# Measuring changes in blood volume fraction during induced gingivitis of healthy and unhealthy populations using hyperspectral spatial frequency domain imaging: a clinical study

**DOI:** 10.1038/s41598-022-23115-x

**Published:** 2022-11-01

**Authors:** Ben E. Urban, Hrebesh M. Subhash, LaTonya Kilpatrick-Liverman

**Affiliations:** Global Technology and Design Center, Colgate Palmolive Technology Center Campus, Piscataway, NJ 08854 USA

**Keywords:** Biological techniques, Biotechnology, Diseases, Health care, Medical research, Optics and photonics

## Abstract

This investigation aimed to quantitatively measure the changes in inflammation of subjects with healthy and unhealthy gums during a period of induced gingivitis. A total of 30 subjects (15 healthy, 15 with gum inflammation) were enlisted and given oral exams by a dental hygienist. Baseline measurements were acquired before a 3-week period of oral hygiene abstinence. The lobene modified gingival index scoring was used for inflammation scoring and hyperspectral spatial frequency domain imaging was used to quantitatively measure oxy- and deoxygenated blood volume fraction at two time points: at Baseline and after 3 weeks of oral hygiene abstinence. We found that abstaining from oral hygiene causes a near proportional increase in oxygenated and deoxygenated blood volume fraction for healthy individuals. For individuals who started the study with mild to moderate gingivitis, increases in blood volume were mainly due to deoxygenated blood.

## Introduction

Gingivitis is a form of gum disease that causes inflammation of the gingival tissue. If left untreated, gingivitis can lead to periodontitis. Periodontitis is an irreversible destructive tissue disease which causes detachment of gingival tissue, deepening of gingival sulcus tissues, formation of periodontal pockets, and bone loss^1–4^. Directly, periodontitis is a major global burden and a link between periodontal disease with other debilitating diseases, such as diabetes, atherosclerosis, cancers, and Alzheimer’s has been suggested^3,5,6^.

An experimental model for investigating inflammation of gingival tissue is induced gingivitis^7^. The induced gingivitis model allows investigators to study the transition of healthy to diseased tissue over short time scales. In the induction phase, gingivitis is created by eliminating all oral hygiene practices over a period of up to 3 weeks. Disruption of oral hygiene causes dental plaque accumulation and affects the microbiome homeostasis of the oral cavity^8–11^. Accumulation of plaque and subsequent disruption of homeostasis causes an inflammatory response of the tissue.

Induced gingivitis has been shown to cause a diverse range of inflammatory responses among individuals^7,12,13^. The inflammation response for subjects generally peaks in the third week of oral hygiene disruption. However, the magnitude and the rate of the inflammatory response can be different between people^7,13,14^. A visual based scoring system is generally used to measure gingival inflammation differences between populations; and in the study described in this manuscript, the lobene modified gingival index (MGI) was employed^15^. Measuring inflammation changes via clinical indices, however, does not provide information on differences in oxy- and deoxygenated blood volumes. In fact, ratiometric differences in oxy- and de-oxygenated blood volume can affect the results of color-based scoring, especially when performed visually. An objective method to quantify changes in inflammatory responses as reflected by changes in blood volumes is, thus, needed to objectively investigate the progression of gingivitis.

Spectral imaging techniques have been successfully applied to map tissue chromophores, such as melanin, blood, and water^16–22^. For mapping tissue chromophores, non-contact imaging techniques are preferable since contact techniques can interfere with chromophore distribution and imaging gives localized structural information. Localized structural information is advantageous for longitudinal investigations or visualizing tissue pathogenesis. Most optical spectral mapping techniques work by measuring the optical absorption at different wavelengths of imbedded tissue chromophores. By using the known absorption spectra of the chromophores at the different wavelengths of light, it is possible to calculate information about chromophore content^16–24^. However, many clinically available techniques calculate ratiometric content of chromophores rather than quantitative measurements. Quantitative measures are difficult because the measured light is a convolution of the scattered and absorbed light. To quantitatively determine chromophore content requires the tissue scattering coefficient to be known or approximated in addition to the absorption coefficient. In quantitative hyperspectral imaging techniques, the tissue scattering coefficient is approximated using theoretical modeling^16,20,25–27^. Hyperspectral spatial frequency domain imaging (Hy-SFDI) is a non-contact spectral mapping technique that directly measures the scattering and absorption coefficients using patterned projections^28–31^. Because the scattering and absorption coefficients are directly measured (deconvolved) for each local tissue region, highly accurate, quantitative measurements of tissue chromophore concentration can be achieved compared to other spectral mapping techniques.

This report investigates the gingival tissue’s inflammatory response over three weeks of oral hygiene abstinence using MGI scoring and a Hy-SFDI system developed to quantify blood volume fraction measurements. The investigation was conducted on groups of healthy people (MGI < 0.5) and people with mild to moderate gingival inflammation (MGI ≥ 2). The large difference in MGI between the healthy and inflamed group was chose to ensure distinction between the two populations using the MGI scoring system. This manuscript will compare oral inflammation measured via MGI scoring and Hy-SFDI assessments in both populations.

## Methods

All experiments in the study were approved by the U.S. Investigational Review Board, Inc. (IRB#U.S.URB2019CP/13) and were conducted at Colgate Technology Campus in Piscataway, New Jersey. All participants provided written informed consent before participating in the study. The clinical study strictly followed the approved protocol without deviation and all methods were performed in accordance with the relevant guidelines and regulations. None of the subjects communicated that they were diabetic or had abnormal sugar levels, high blood pressure, or heart disease.

### Participant recruitment

Participants were respondents to the Colgate Technology Campus (Piscataway, NJ) clinical site’s advertised announcement for a clinical study. Participants lived in the New Jersey area and were selected based on the inclusion and exclusion criteria defined in the below sections.

### Inclusion criteria

A summary of participants in the clinical study is shown in Table 1. The criteria for inclusion were as follows: male or female, 18–65 years of age; subject in good general health; available for the duration of the study; willing to sign the consent form; at least 20 natural teeth; Modified Gingival Index (MGI) < 0.5 or MGI ≥ 2 for the healthy and unhealthy groups, respectively. 29 subjects completed the study. One person dropped out for personal reasons. There were no adverse events reported.

**Table 1:** Demographic data table of the subjects participating in the clinical study at baseline.

Variable	Analyzed group		Total group	
	Healthy (N = 9)	Unhealthy (N = 9)	Healthy (N = 15)	Unhealthy (N = 15)
Average age	43.3 (3.6)	41.4 (4.1)	40.5 (2.52)	43.3 (3.07)
Male	3	6	6	8
Female	6	3	9	7
Average incisor baseline MGI	0.194 (0.018)	2.833 (0.050)	0.142 (0.027)	2.417 (0.043)

Data are not representative of the full month MGI scores. The MGI scores capture the measurements from the upper and lower incisors (tooth # 7–10 and 23–26) and are expressed as mean (standard error of the mean) values.

*MGI* modified gingival index.

### Exclusion criteria

Subjects were excluded if they met the following criteria: the subject had medical conditions which required premedication prior to dental visits/procedures; had knowledge of impaired salivary function; used medication that could affect salivary flow; had used anticonvulsants, antihistamines, antidepressants, sedatives, tranquilizers, antibiotics, antimicrobial, anti-inflammatory medication or daily analgesics within 30 days prior to the start of the study or started such intake during the course of the study; had an ongoing use of medications known to affect the gingival tissues (i.e. calcium channel blockers, phenytoin, cyclosporine); had a history of infectious disease (i.e. Hepatitis B or C, HIV, oral herpes); had contagious illness (i.e. upper respiratory infection, oral herpes, sinusitis); had dentures; had < 20 natural teeth; had periodontal pockets (> 4 mm deep); had carious lesions requiring immediate restorative treatment; had evidence of periodontal disease; had history of allergy to consumer or personal care products or dentifrice ingredients as determined by the dental professional monitoring the study; was pregnant or nursing; was participating in another oral clinical study or test panels that required oral ingestion or testing of a product within a 1 month prior to entering the study; had received dental treatment during the study dates; was a smoker or used tobacco or e-cigarette products; had braces or aligners.

### Study visits and clinical procedures

Participants were first screened to assess whether they met the inclusion/exclusion criteria. At the screening, participants’ demographics and medical history were recorded, followed by an oral examination including a gingival assessment (described below in the “Health determination” section). Assessment results were recorded. After signing an Informed Consent Form, being provided a Privacy notice, and meeting all inclusion requirements, subjects were enrolled in the study. 30 participants were enrolled in the study (15 with MGI < 0.5 and 15 with MGI ≥ 2). After enrollment, these participants received a prophylaxis cleaning to remove all plaque and calculus. They were given a soft-bristled toothbrush and sodium fluoride (0.76%) containing toothpaste and instructed to brush twice a day for 2 min for 14 days. After this 14 day period, the participants returned for their baseline visit. They were asked to abstain from brushing 12 h prior to the baseline and for every successive visit to the clinical site. At each visit during the induced gingivitis stage (2, 7, 14, and 21 days post Baseline), participants received full oral soft tissue examinations and assessments of gingival inflammation (MGI). Hyperspectral Spatial Frequency Domain Imaging measurements were only conducted at Baseline and at the Day 21 visit. After the Baseline visit, the participants were instructed to abstain from performing any oral hygiene (i.e. toothbrushing, use of mouthwash, flossing, etc.) for 3 weeks. After the 3 weeks visit, subjects were instructed to return to their normal oral hygiene regimen, brushing twice a day for 2 min for 14 days with the provided fluoride containing toothpaste. After the conclusion of the 14-day period, the subjects returned to the clinic for a final clinical assessment of MGI and health.

### Health determination

All panelists were given an oral exam by a dental hygienist. The examination included evaluation of the soft and hard palate, gingival mucosa, buccal mucosa, mucogingival fold areas, tongue, sublingual and submandibular areas, salivary glands, and the tonsillar and pharyngeal areas. The panelists also completed a Medical History form to determine if they met the inclusion/exclusion requirements. Panelists that met the inclusion criteria were classified as healthy or unhealthy based on MGI scoring. Chosen panelists were not known to be susceptible to inflammation other than what would be driven by having poor oral hygiene habits.

### Modified gingival index scoring

**Figure 1:**
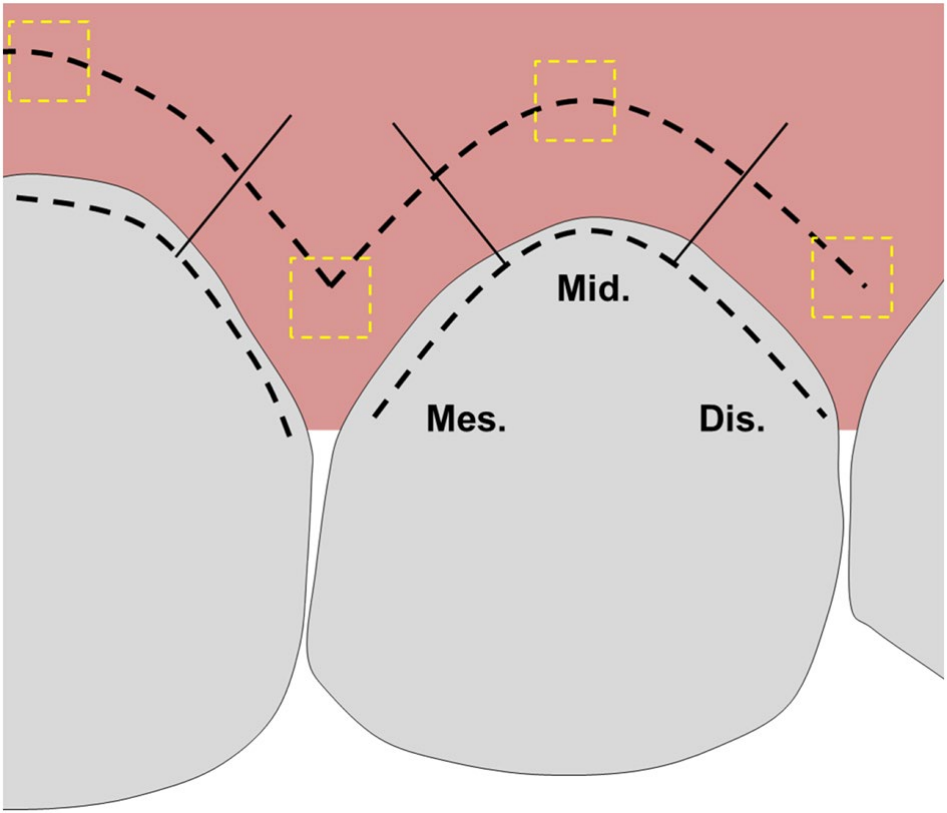
Cartoon showing the MGI scoring and Hy-SFDI data collection sites. The black dashed line shows the location of MGI scoring and the solid black line shows the division of the mesial, middle, and distal sections of the tooth. MGI was scored separately for each of the tooth sections. The yellow dashed boxes show the approximate locations where the Hy-SFDI data was collected. A total of 14 Hy-SFDI were collected (7 from the upper incisors and 7 from the lower incisors).

Non-invasive MGI scoring was chosen to prevent bleeding and disturbing plaque growth during the longitudinal investigation. MGI was scored at the mesial, middle and distal locations on the buccal tooth surface for the upper and lower incisors (8 total teeth), as shown in Fig. 1. The MGI of the sites were recorded at baseline (normal hygiene) and again at 2, 7, 14, and 21 days of abstaining from brushing (induced gingivitis). A description of MGI scoring is detailed in Table 2^15^. Since this report captures the changes in oxygen and de-oxygenation at only two time points: Baseline and at Day 21, only the MGI data at these two timepoints will be presented in this manuscript. See all of the MGI data in the Supplemental Information.

**Table 2:** Description of the modified gingival index scoring system.

Score	Marginal gingival index (MGI)	Visual	Health classification
0	Absence of inflammation	Normal color and morphology	Healthy
1	Mild inflammation	Slight change in color and morphology in localized gingival margin	Healthy
2	Mild inflammation	Slight change in color and morphology of majority/all portions of gingival margin	Unhealthy
3	Moderate inflammation	Red color, edema, morphological swelling	Unhealthy
4	Severe inflammation	Red color, edema, ulceration, spontaneous bleeding, and morphological welling	Unhealthy

### Hy-SFDI image acquisition and processing

Functional blood information was measured using a hyperspectral spatial frequency domain imaging (Hy-SFDI) system^31,32^. The location of data collection is shown in Fig. 1. Example images of the data are shown in Supplementary Fig. S1. The details of the system and data processing have previously been reported^31^. Briefly, a compact LED projector (P2-A, AAXA Technologies) was used to projector light through a polarizer and onto the oral cavity surface. The projected light was patterned at spatial frequencies of 0 and 0.2 mm^−1^ for the low and high frequency components, respectively. The high frequency projected pattern was twice phases shifted by $2\pi /3$ to cover the entire imaging area. A 16 channel hyperspectral camera (MQ022HG-IM-SM4X4-VIS, Ximea) placed 34 cm from the sample surface was used to image backscattered light from the tissue surface. Backscattered light first passed through a cross-polarizer to remove specular reflection.

Hyperspectral images were captured at each illumination spatial frequency and phase. Additionally a background images was acquired to account for camera and ambient noise conditions. A total of five images were acquired for Hy-SFDI (1—0 mm^−1^, 3—0.2 mm^−1^, 1—background). A total acquisition time of 0.3 s was necessary to capture all images for Hy-SFDI. Due to significant spectral cross-talk, only 12 channels (502, 512, 524, 538, 562, 573, 586, 598, 611, 620, 632, 636) of the hyperspectral images were used for chromophore calculation. Captured images were first corrected using a company provided correction matrix. A modified open-source SFDI program was used in addition to a custom generated Monte Carlo look-up table for determining the absorption and scattering coefficients. Finally, the absorption coefficient at each wavelength was used to quantitatively map volumetric blood concentrations in the oral cavity (see Supplementary Fig. S1).

Subjects were given a cheek retractor to expose their gingival tissue for imaging. After the gingival tissue was well exposed, the subjects placed their head in a chin rest with forehead support for stability. Hy-SFDI images were then acquired for analysis. The acquisition time was approximately 300 ms. The imaging protocol was executed at baseline (normal hygiene) and again after 3-weeks of abstaining from brushing (induced inflammation). The measurements were taken only at these two time points because the inclusion of the Hy-SFDI system was for exploratory purposes in this study.

### Exclusion of panelists from data

One panelist dropped out of the study before the 3-week imaging session. Their data was not used in the evaluation of this report. 11 of the Hy-SFDI sets (6 at baseline and 5 at the 3-week imaging session) presented frequency artifacts due to mis-matched calibration and imaging distances. Therefore, Hy-SFDI data with frequency artifacts were also removed. Data from the remaining 18 panelists were used in the evaluation and presented in this report.

### Longitudinal measurement of oxy- and deoxy-blood volume fraction

After Hy-SFDI image acquisition, images were used to generate oxy- and deoxygenated blood volume fraction maps of the gingival tissues of the oral cavity of the 18 subjects without Hy-SFDI frequency artifacts^33^. The maps were then used to select a 10X10 pixel area (approximately 1 × 1 mm^2^) on the gingival tissue approximately 1 mm above the central crest of the upper incisors and 1 mm below the trough of the lower incisors. The area was then averaged to get the oxy- and de-oxygenated blood volume fraction for the selected region. The base and 3-week time point images for each subject were co-registered to acquire the blood volume fraction from the same location at the different time points.

### Longitudinal measurement of modified gingival index score

To compare MGI score and Hy-SFDI measurements, the anterior facial MGI measurements of the incisors were averaged and used for analysis.

### Statistical analysis

For MGI measurements, the mesial, middle, and distal MGI measures from the eight incisors (four upper, four lower) were averaged for each person to give an average MGI score of (1) the upper incisors, (2) the lower incisors, and (3) total incisors. Each panelist received an average score, and the standard error of the mean was calculated for each group (healthy and unhealthy). For blood volume measurements, the sampling method is described in the “Longitudinal measurement of oxy- and deoxy-blood volume fraction” section. Oxy and Deoxy-blood volume fractions from the upper incisors and lower incisors were averaged to give an Oxy and Deoxy-blood volume fraction average of the total incisors. Each panelist received an average score, and the standard error of the mean was calculated for each group (healthy and unhealthy).

## Results

### Modified gingival index dynamics

**Figure 2:**
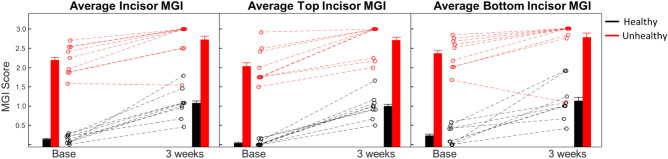
MGI scores of the incisors from healthy and unhealthy panelists at baseline and 3 weeks. (Left) The average MGI of the incisors. (Center) The average MGI of the top incisors. (Right) The average MGI of the bottom incisors. Data from each of the individual panelists is indicated with a dashed line to show how the individual score changed. Bar graphs show the average trend of the healthy (black) and unhealthy (red) groups. Error bars on the bar graph are the Standard Error of the Mean for each group.

MGI scores of panelists at baseline and after 3 weeks of abstaining from oral hygiene are plotted in Fig. 2. Clear changes in MGI were observed for the healthy group, whereas the unhealthy group had a smaller MGI change. For the healthy group, the average MGI score increased from 0.14 ± 0.02 to 1.07 ± 0.07 over the 3 week period, confirming the inflammatory response. For the unhealthy group, the average MGI score increased from 2.19 ± 0.07 to 2.73 ± 0.08. Changes for both groups showed an increase in inflammation according to MGI score, but the healthy group showed a larger overall increase. Changes in MGI score for both groups for the top and bottom jaw had similar trends as compared to the averaged MGI score, however a single panelist did experience a decrease in MGI score for the lower incisors. Changes in MGI score were found to be statistically significant for both groups (see Supplementary Table S1).

### Blood volume fraction dynamics

**Figure 3:**
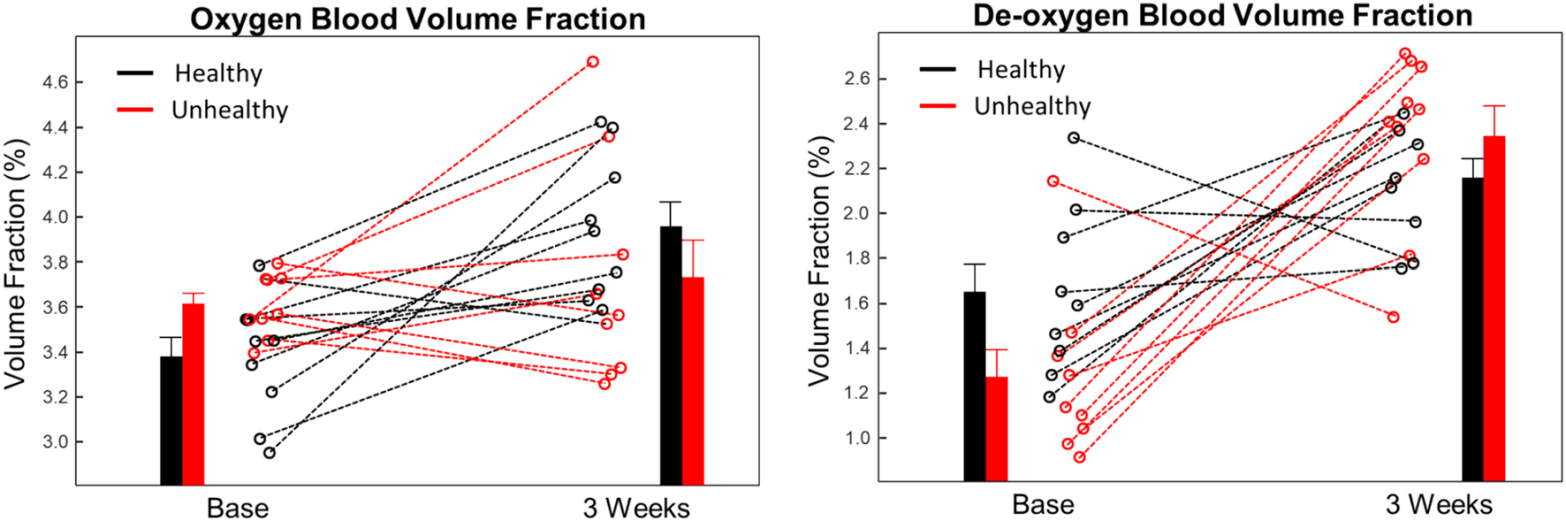
Spatial frequency domain blood volume measurements of healthy and unhealthy panelists at baseline and 3-weeks. (Left) Oxygenated blood volume fraction for each panelist. (Right) De-oxygenated blood volume fraction for each panelist. Data from each of the individual panelists is indicated with a dashed line. Solid lines show the average trend of either the healthy (black) or unhealthy (red). Error bars are standard error of the mean.

Figure 3 shows the blood volume fraction dynamics for the panelists. Similar to MGI scores, oxygenated and de-oxygenated blood volume fraction changes were observed in healthy and unhealthy groups. In general, changes in the blood volume fraction corroborated MGI measurements for the healthy group. Overall blood volume fraction increased for both healthy and unhealthy groups during three-weeks of non-brushing. The healthy group presented a larger and statistically significant increase in average oxygenated blood volume fraction (~ 17%) compared to the unhealthy group, whereas the unhealthy group had a larger and statistically significant increase in de-oxygenated blood volume fraction (~ 85%) (see Supplementary Table S1). On an individual level, a majority of the panelists in the healthy group showed an increase in both oxy- and de-oxygenated blood volume fractions. For oxygen blood volume fraction, the unhealthy group had a diverse response; five panelists presented a decrease and four panelists increased or remained the same. In contrast, blood oxygen fraction increased for all panelists in the healthy group. The unhealthy group had a less diverse increasing trend for de-oxygenated blood volume when compared to the oxygenated blood volume response.

### Adverse events

No adverse events were reported by any of the panelists during the period of the investigation.

## Discussion

Results show that both MGI and Hy-SFDI measured inflammatory responses of panelists in both the healthy and unhealthy groups after 3 weeks of oral hygiene abstinence. However, Hy-SFDI demonstrates that the blood component response is apparently different on average for the two groups. Among the blood components measured, de-oxygenated blood volume fraction for the healthy group and oxygenated blood volume fraction for the unhealthy group were not found statistically significant. Changes in MGI had low to moderate correlation with blood volume fraction components for the healthy group, but presented high correlation with all blood volume fraction components in the unhealthy group (see Supplementary Table S2).

MGI is a visual scoring scale, defined in Table 2. MGI evaluates the level of oral tissue inflammation and gingival health. Oral tissue inflammation is known to occur in healthy populations with immediate withdrawal of oral hygiene. Induced gingivitis has been shown to peak after 3 weeks of oral hygiene abstinence. Previous induced gingivitis investigations show a diverse inflammation response composed of low and high responders^7,12–14,34,35^. However, comparing MGI scoring and quantitative blood volume measurements during induced gingivitis of healthy people and populations with mild to moderate gingival inflammation has not been investigated.

Immediate withdrawal of oral hygiene leads to changes in gingiva tissue MGI score of healthy subjects as well as those with gum inflammation. Panelists in both groups showed an increase in average MGI scoring, consistent with what has been observed for experimentally induced gingivitis. However, the unhealthy group had a less pronounced average MGI increase in response to induced gingivitis. Both groups had a prophylaxis cleaning and followed a designated oral cleaning routine (described in the “Study visits and clinical procedures” section) before the baseline measurements. The professional cleaning and hygiene routine reduced gum inflammation for the unhealthy group (average ΔMGI =  − 0.52) and had no significant change for the healthy group (average ΔMGI =  + 0.01). The average MGI score at Baseline for the subjects with gum inflammation still, however, fell in the mild to moderate range (Table 2). The application of prophylaxis cleaning with 2x/day brushing thus only slightly mitigated gum inflammation experienced by this population. During the 3-week brushing abstinence, the unhealthy panelists’ MGI score returned to the original pre-brushing routine homeostasis.

Hy-SFDI functional blood volume measurements showed different inflammatory responses for the healthy and unhealthy groups. The healthy group had a larger increase in oxygenated blood volume at the 3-week time point. In the unhealthy group, the blood volume increase was mainly due to de-oxygenated blood. Increases in de-oxygenated blood volume cause tissue to appear darker, whereas increases in oxygenated blood cause a redder appearance to tissue^36^. The impact of blood oxygenation status on color may have led to an apparent smaller MGI reduction for the subjects with mild to moderate gingivitis at Baseline. Both healthy and unhealthy groups showed increased blood volume fraction after 3 weeks of abstaining from oral hygiene. The average blood volume fraction was similar between both groups after 3 weeks of oral hygiene abstinence. However, the de-oxygenated blood volume fraction increase was more pronounced and statistically significant for the unhealthy group (see Supplementary Table S1). Therefore, either the tissue-oxygen demand was higher for the unhealthy population or vasculature created by angiogenesis, which occurs in chronically inflamed tissues, feeds the tissue with a higher percentage of de-oxygenated blood^37,38^.

The current investigation demonstrates an apparent quantitative difference in gingival blood functional response of healthy and gingivally unhealthy groups due to withdrawal of oral hygiene. Strength of correlation between changes and MGI and blood volume fraction components differed depending on oral health. Large variation in response to experimental gingivitis has been observed in previous investigations. Therefore, to confirm the blood functional response difference between the two groups, a larger population is needed with measurements acquired at more time points. In addition, the current investigation is limited to the front of the incisors. Limited field-of-view also limits better understanding of inflammatory response, which can differ regionally in the oral cavity. Incorporating 3D depth measurements into the current Hy-SFDI system will greatly improve diagnostic capabilities.

## Supplementary Information

Supplementary Information.

## Data Availability

The datasets generated and/or analysed during the current study are not publicly available due to company confidentiality policy but are available from the corresponding author on reasonable request.
