# Supplementary material for: Measuring changes in blood volume fraction during induced gingivitis of healthy and unhealthy populations using hyperspectral spatial frequency domain imaging: a clinical study
Source: Sci Rep. 2022 Nov 1;12:18357. doi: 10.1038/s41598-022-23115-x (PMC9626635; doi:10.1038/s41598-022-23115-x)
Supplement: Supplementary file 1 — Supplementary Information. [file 41598_2022_23115_MOESM1_ESM.docx]

**Supplement Information:**

**Measuring changes in blood volume fraction during induced gingivitis of healthy and unhealthy populations using Hyperspectral Spatial Frequency Domain Imaging: A clinical study**

Authors: *Ben E. Urban, Hrebesh M. Subhash, and LaTonya Kilpatrick-Liverman

*Global Technology and Design Center, Colgate Palmolive Technology Center Campus, Piscataway, NJ 08854, USA*

**ben_urban@colpal.com*

**1. Example Blood Volume Fraction Maps of Panelists**

To determine quantitative changes in blood volume fraction components we used Hy-SFDI. Maps of the blood volume fraction percentage of oxygenated (HbO) and deoxygenated (HbR) blood components at baseline and 3-weeks after oral hygiene abstinence are shown in SI Figure 1. On average, healthy panelists presented an increase in both HbO and HbR volume fraction percentage, however only HbO was found to be statistically significant for the group (SI Table 1). Unhealthy panelists presented only a minor average HbO increase, though the increase was not statistically significant (SI Table 1) and was highly diverse among panelists (Figure 3). On the other hand, unhealthy panelists showed a statistically significant increase in HbR volume fraction percentage, which was near uniform across the unhealthy group.


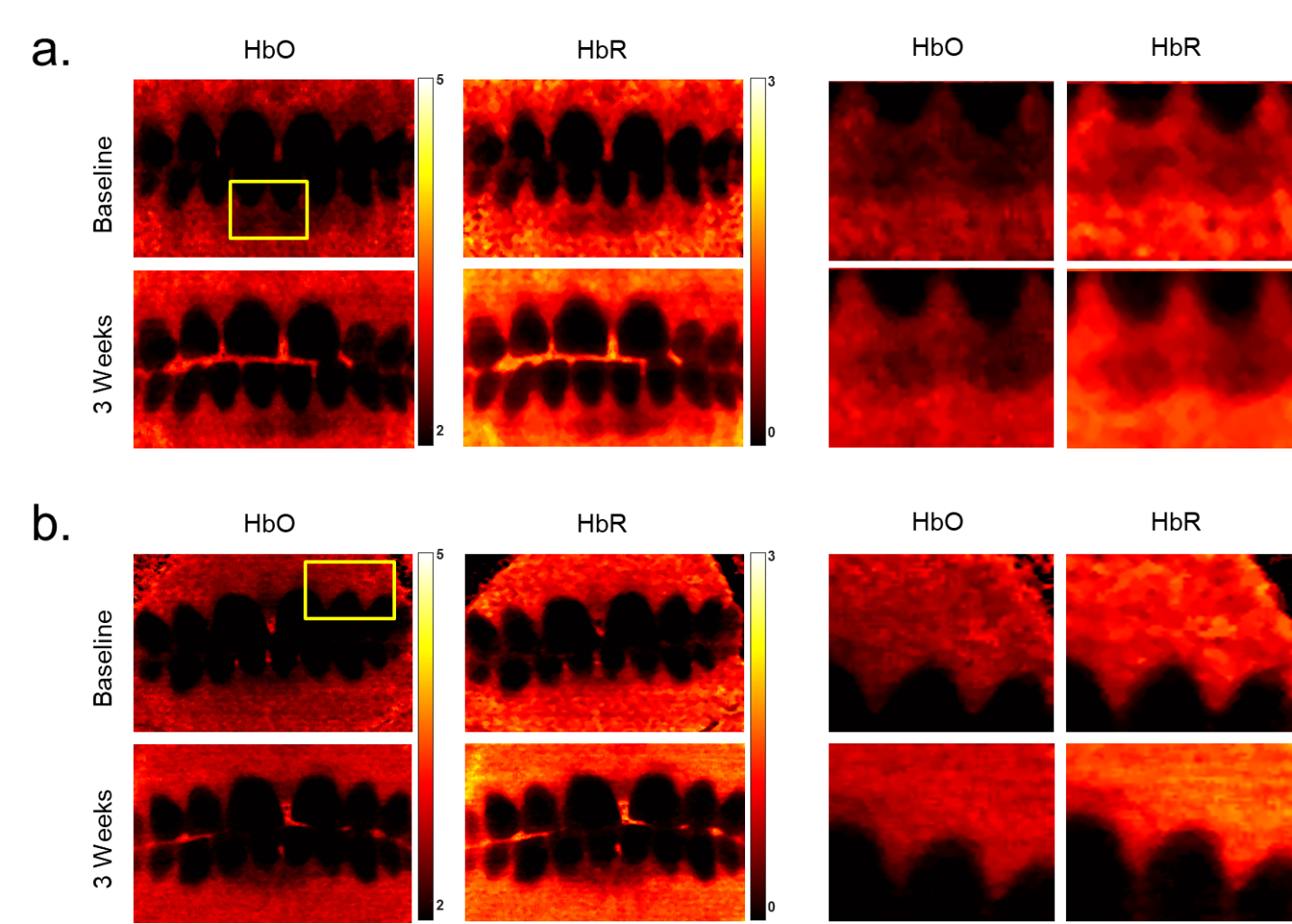


**Figure S1.** Oxygenated and de-oxygenated blood volume fraction percentage map of (a) a *healthy* panelist and (b) and *unhealthy* panelist at baseline and 3-week time points. The yellow box in the HbO image shows the location of the zoomed images displayed on the right of the figure. The blood volume fraction is displayed using an intensity map as indicated by the scale to the right of the images.

**2. Statistical Evaluation**

To determine statistical significance level of changes in MGI and Hy-SFDI measurements from baseline to the 3-week time point, we used a paired-sample, left side t-test (Table S1). The null hypothesis of the test is that the pairwise difference between the baseline and 3-week time point has a mean equal to zero at the 1% significance level. HbO blood volume fraction of the unhealthy group and HbR blood volume fraction of the healthy group were there only tested parameters that were not statistically significant. Given the limited number of panelists in the groups in this clinical study, it cannot be ruled out that there is still a relation between these components.


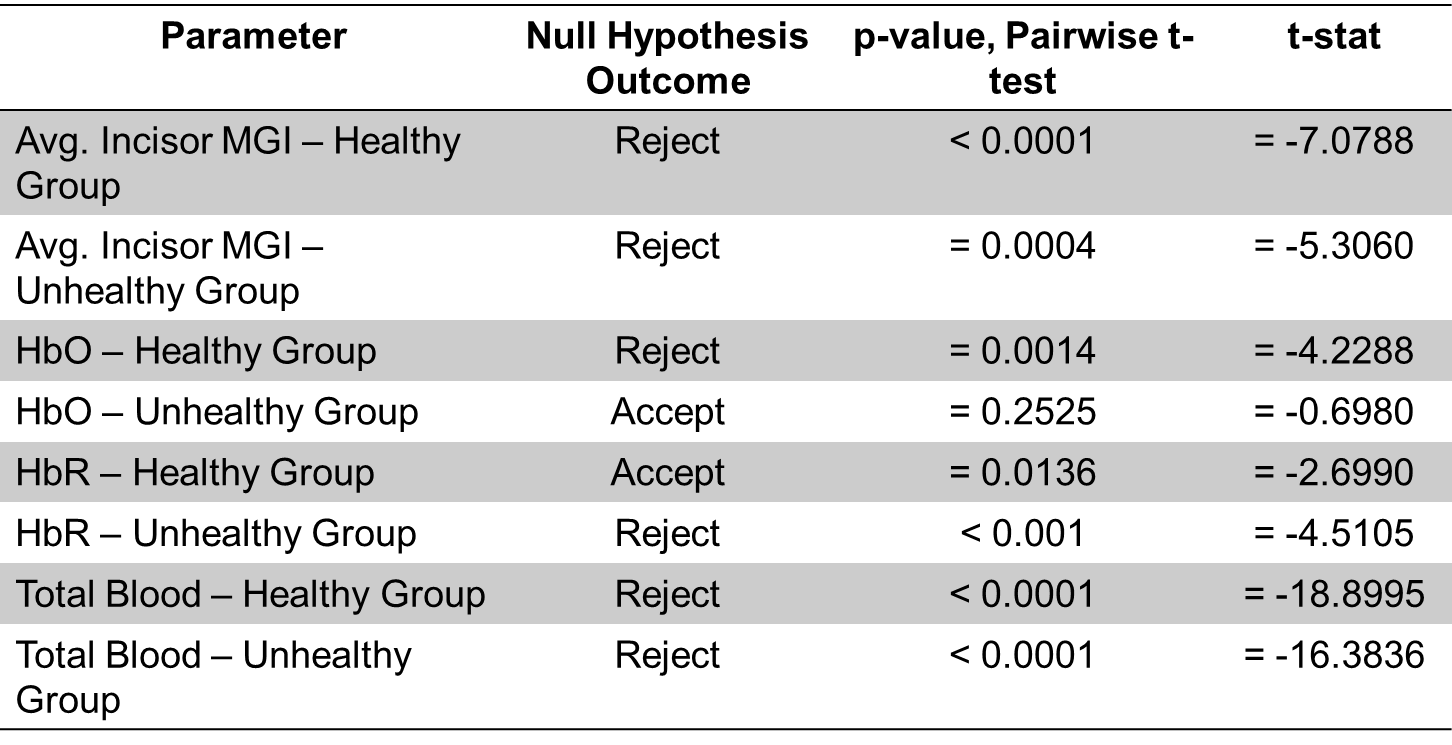


**Table S1.** Statistical results of MGI and Hy-SFDI measurements. MGI showed a significant difference (rejection of null hypothesis) for both healthy and unhealthy groups. For Hy-SFDI parameters, the HbO volume fraction percentage of the unhealthy group did not significantly change in the time period. Similarly, the HbR volume fraction percentage of the healthy group did not significantly change. All other components were statistically significant. The t-stat parameter is the raw t-test value.

To determine if there was a linear relationship between changes in clinical MGI scoring and quantitative Hy-SFDI blood volume fraction, we calculated the Pearson Correlation Coefficient (SI Table 2). Notably, MGI scoring is subjective and will likely depend on the clinician. In our investigation, a single clinician was used for MGI scoring. In the interpretation of the data, the sign indicates a positive or negative relationship and the strength of correlation was determined to be Very Low (0 < r ≤ 0.19), Low (0.2 ≤ r ≤ 0.39), Moderate (0.4 ≤ r ≤ 0.59), High (0.6 ≤ r ≤ 0.79), or Very High (0.8 ≤ r ≤ 1.0). In summary, for the healthy group, moderate to low correlation was found between changes in MGI and blood parameters. On the other hand, for the unhealthy group, high correlation was found between changes in MGI and blood parameters.


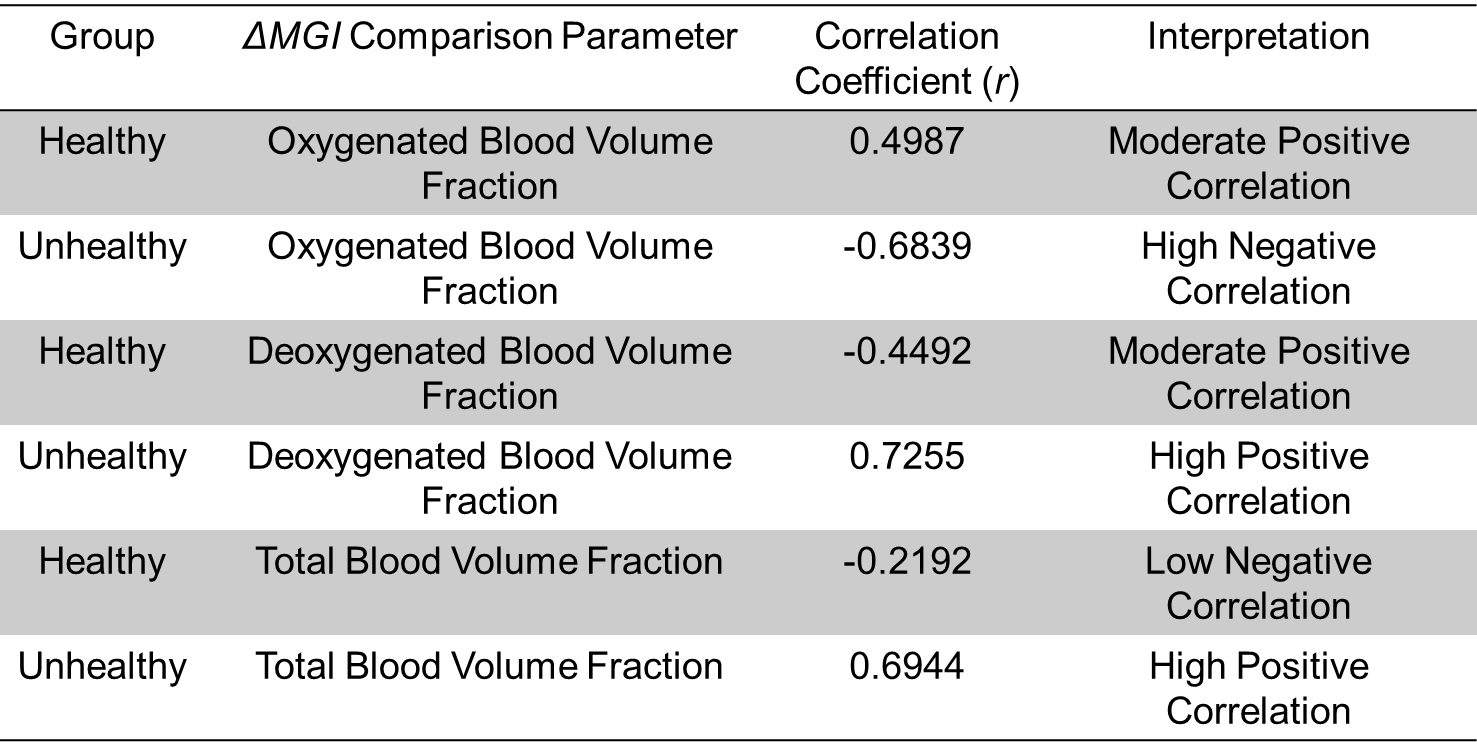


**Table S2.** Correlation coefficient values of the change in MGI vs change in Hy-SFDI blood volume fraction measurements.
